# Geographical Proximity and Community Health Centres: A Sustainable Solution for Denmark

**DOI:** 10.3390/ijerph23020173

**Published:** 2026-01-30

**Authors:** Nanna Finne Skovrup, Malene Freudendal-Pedersen

**Affiliations:** Department of Sustainability and Planning, Aalborg University, 2450 Copenhagen SV, Denmark; mfp@plan.aau.dk

**Keywords:** accessibility, 15-min city, social and cultural sustainability, rural healthcare, geographical proximity

## Abstract

**Highlights:**

**Public health relevance—How does this work relate to a public health issue?**

- Geographical proximity, encompassing both physical distance and relational access, influences patient well-being and health equity, particularly in rural and island contexts.
- Everyday geographies and small-scale mobility patterns are crucial in determining whether residents can effectively access and utilise healthcare services.

**Public health significance—Why is this work of significance to public health?**

- The findings show that integrating sectors such as health services, urban planning, and social policy is crucial to closing accessibility gaps in sparsely populated areas.
- Additionally, social and cultural sustainability plays a vital role in effective healthcare, emphasising the significance of local social networks, cultural traditions, and community cohesion.

**Public health implications—What are the key implications or messages for practitioners, policymakers and/or researchers in public health?**

- Rural and island healthcare systems require patient-focused planning that accounts for transport challenges, local practices, and organisational barriers, even when services are available.
- Island municipalities offer lessons in proximity-based, multifunctional service models that can inform equitable health planning in settings beyond rural areas.

**Abstract:**

The Danish healthcare system has transitioned from a decentralised municipal hospital model to a centralised structure dominated by large, specialised hospitals. While this shift has improved efficiency and healthcare quality in some respects, it has also created challenges in terms of accessibility, patient mobility, and sustainability. Community health centres represent a strategic response to these issues by decentralising essential healthcare services and reintroducing geographical proximity as a core principle of healthcare. In this article, we propose drawing on the 15-min city concept to discuss how accessibility and spatial equity should be integrated into the planning of community health centres as platforms for active living and strong communities. We argue that proximity and accessibility in healthcare can benefit from a broader view of mobility and a focus on developing active, independent mobility systems. Data from semi-structured interviews with patients and professionals at 11 community health centres and three regions empirically demonstrate this. The 15-min city concept can lead to a reduction in travel time and improve accessibility and proximity for older adults.

## 1. Introduction

The growing proportion of older adults in Western societies has led to an increase in multimorbidity, resulting in a greater demand for healthcare services. In Denmark, the percentage of the population aged 65 and over is projected to reach 35.3% by 2060, and this trend is anticipated to persist [1]. A significant policy response has been the establishment of six large, highly specialised hospitals, strategically centralised to improve healthcare quality. However, this centralisation has led to longer travel distances for patients, particularly those in rural areas, resulting in challenges related to accessibility and mobility [2].

**Figure 1:**
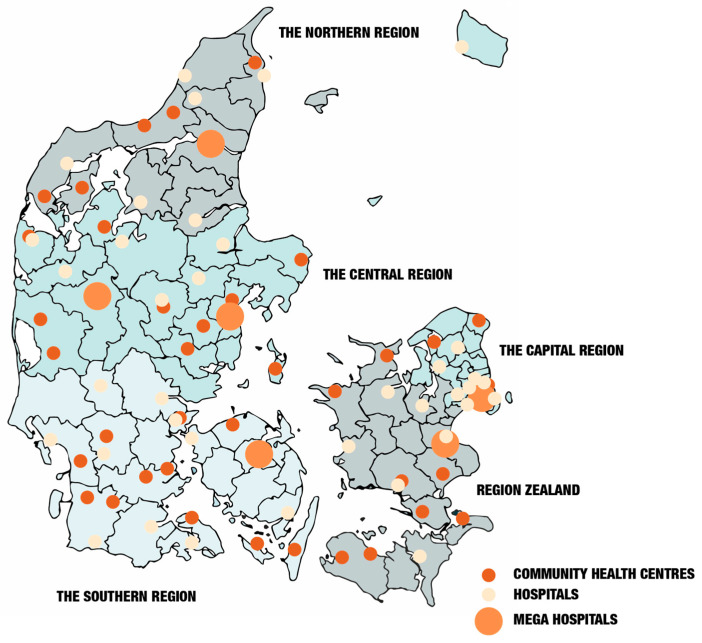
The Danish healthcare landscape is marked by dots representing mega hospitals, community health centres, and hospitals. Please note that the research was conducted before the establishment of Region East, which includes Region Zealand and the Capital Region. The regions are indicated with grey and blue colours [11].

To tackle these challenges, the Danish government, in conjunction with primary (municipal) and secondary (regional) healthcare providers, has established 36 cross-sectoral community health centres (see Figure 1). These decentralised healthcare hubs aim to improve accessibility by offering essential services such as general practice, physiotherapy, health visiting, blood sampling, radiology, and outpatient care. By prioritising geographical proximity, the idea behind these centres is to enhance access to healthcare, facilitate patient mobility, and contribute to a more sustainable healthcare system [3]. Moreover, in some cases, this seems to be working, especially when they are placed in the former hospitals that were closed due to the centralisation into mega hospitals. These former hospitals were built in the city centre at a time when access was not primarily based on private cars. In some places in Denmark, however, new buildings were constructed for the community health centres located on the outskirts of the city, making it difficult for older adults who are unable to drive to access them. What is missing in the planning of community health centres is attention to the benefits of accessibility and spatial equity [4,5]. Cities designed to encourage active mobility and accessibility foster lifestyles that are beneficial for overall physical and mental health through active and sustainable living [6]. This article discusses the importance of a strategic planning concept for community health centres to ensure their intended function. It examines how community health centres, as a proximity-based healthcare model, can benefit from integration with the planning concept of the 15-min city. This will enhance the sustainability and align with Denmark’s broader sustainability and healthcare objectives. First, the centralisation and decentralisation of the healthcare system in Denmark are outlined, and their relationship to objectives for sustainability and healthcare is discussed. Following this, the 15-min planning concept is presented as a pathway to integrate these objectives. Based on this, interviews with healthcare professionals and patients are used to exemplify the need for a more holistic approach to community health centres. In the concluding discussion, we emphasise the importance of incorporating active mobility into planning as an essential component of everyday living for healthier lives. This also entails a greater emphasis on the cultural and social aspects of sustainability.

## 2. The Centralisation and Decentralisation of Healthcare in Denmark

Since the post-World War II welfare reforms, the Danish healthcare system has undergone significant structural changes. Established initially as an egalitarian system with numerous municipal hospitals, the total number has sharply declined from 117 in 1980 to just 35 by 2020 [7,8]. The launch of six mega-hospitals in 2009, designed as multi-campus healthcare facilities, marks a crucial turning point in the centralisation of healthcare in Denmark [9], see Figure 1. The last mega-hospitals are expected to open by the end of 2027. While this restructuring aimed to enhance efficiency and specialisation, it has also increased the gap between patients and healthcare services, particularly affecting those living in rural areas.

An argument in favour of mega-hospitals was their ability to minimise patient and professional transport among various facilities, leading to more efficient resource usage [10]. What was never considered in the planning of these mega-hospitals was the impact on transportation for employees, patients, and relatives. This centralisation has heightened transportation demands, particularly in areas with limited public transportation options. Research reveals that 17% of emissions from the Danish healthcare sector stem from operational activities and transportation [10], raising concerns about environmental impact and patient accessibility.

In response to these challenges, the Danish government introduced the ‘Make Denmark Healthier’ proposal in 2022 [12]. This policy initiative aims to reduce healthcare-related CO_2_ emissions by 75% by 2030, primarily through sustainable building operations and transportation reductions [10]. The proposal emphasises the necessity of integrating environmentally sustainable practices within healthcare infrastructure, including energy-efficient facility design and improved public transportation links to healthcare sites.

Comparative international research highlights the importance of transport-related emissions within healthcare systems. A study conducted in the Netherlands found that transportation is responsible for 5.3% of total healthcare emissions [13]. Technological advancements, such as telemedicine and digital health solutions, offer opportunities to reduce these emissions while also enhancing healthcare access and efficiency [11,14].

The location of healthcare facilities is crucial for patient access and healthcare equity. For many people, especially those who rely on public transportation, accessing healthcare services can be a significant challenge [15,16]. Placing community health centres near public transport hubs and city centres enhances accessibility, reducing the need for long-distance travel.

However, the geographical placement of newly established community health centres varies. Many repurposed centres occupy former hospital sites, benefiting from their historically central locations [11]. In contrast, some newly constructed centres have been situated in peripheral areas, necessitating adjustments in public transport infrastructure to ensure accessibility. For instance, in Elsinore, in the Capital Region, the opening of a new community health centre prompted modifications to the local public transport network to accommodate patients and healthcare professionals [17]. This underscores the importance of integrated transport and healthcare planning within a proximity-oriented planning framework, ensuring that healthcare services are not only geographically proximate but also functionally accessible.

### 2.1. Social and Cultural Sustainability, Healthcare, and Local Accessibility

While discussions on healthcare sustainability often focus on economic efficiency and environmental impacts, the social and cultural aspects are just as vital [18,19]. Social sustainability encompasses a healthcare system’s capacity to ensure equitable access, foster trust, and enhance community resilience over time. Cultural sustainability, on the other hand, acknowledges that healthcare is rooted in local norms, values, and personal experiences, and that services must be aligned with these contexts to remain relevant and trustworthy [20,21].

Local accessibility is essential for both types of sustainability. For patients, being able to access care nearby is not just convenient but also helps preserve daily routines, social connections, and sense of place [22]. When services are centralised in distant locations, patients in rural or island communities may face longer travel times and experience social dislocation, where the healthcare journey interrupts family and community bonds [23].

Furthermore, accessibility goes beyond just physical distance. It includes relational and cultural factors, such as the ability to connect with familiar healthcare professionals, communicate using shared linguistic and social codes, and feel recognised during healthcare encounters [24,25]. From this perspective, healthcare systems that reduce local presence risk damaging social cohesion and trust, which are vital resources for long-term sustainability.

The challenge is not just relocating services geographically but maintaining care practices that are socially and culturally integrated into local communities. This is especially important in peripheral areas, where closing small clinics or merging services can have a more significant impact on vulnerable populations, exacerbating existing inequalities [26].

This connects to a stronger focus on everyday life as the starting point when considering the planning of healthcare facilities. Historically, the everyday has often been undervalued in planning, typically viewed as trivial or insignificant. However, for most people, daily life involves a web of interconnected activities, where mobility and accessibility are integral to the sense of life quality [27]. The concept of everyday life is both a research field and an epistemological starting point that provides a distinctive lens on the object of study. The everyday perspective offers means of understanding how specific practices are generated and sustained, thereby also shaping the cultures, politics, and planning of systems they inhabit [27,28]. By centring everyday life, community health centres and proximity become an integrated part of sustaining and maintaining communities. It calls for a different approach to planning where economic rationality is not enough, but focus also needs to be on cultural and social sustainability.

### 2.2. The 15-Min City as an Example of Proximity-Oriented Planning

The planning concept of the 15-min city has gained significant international momentum in recent years due to its potential to reshape urban environments in response to pressing global challenges, including climate change, social inequality, and the impacts of the COVID-19 pandemic. Leading global organisations, including the IPCC, C40 Cities, and UN-Habitat, are actively advancing international cooperation on its implementation [29,30].

First introduced by Carlos Moreno in 2016, the 15-min city proposes a transformation of urban design to ensure that residents can access all essential services and amenities—such as work, education, healthcare, shops, and leisure—within a 15-min walk or bike ride from their homes [31,32]. The precise duration denoted by the “15-min” label is not inherently fixed; the model is adaptable and may be conceptualised as a 5-, 10-, 20-, or even 30-min city, depending on local context and planning objectives [33]. However, the temporal dimension of the concept remains central, as it marks a paradigmatic shift in urban planning from a traditional distance-based model—where spatial separation is quantified in metres or kilometres—towards a time-based approach, where accessibility is measured in minutes. This way of thinking aligns with everyday life routines, where the distance to the supermarket or cinema, for instance, is most often explained as being 10 min’ walking or 5 min’ car driving. This reconceptualisation foregrounds the human scale of urban experience and emphasises transportation as more than just a matter of covering distance between activities [34]. Incorporating the subjective experience of time into urban planning necessitates an understanding of the dynamic rhythms of urban life. The same space may serve vastly different functions depending on the time of day or week; for example, a public square may be lively and populated during daylight hours and deserted at night. A community health centre can serve as a community space both within and outside opening hours. Consequently, urban functions should be viewed as temporally fluid rather than fixed, and they evolve in response to the temporal rhythms of city life, thereby enhancing the efficiency, inclusivity, and vibrancy of urban spaces. In this context, active mobility is understood as a means to enhance opportunities for social interaction, both in places and during movement [31].

With the flexible time frame, the planning concept can be adapted to different urban contexts, ranging from 5 to 20 min, depending on local conditions. In large cities, the approach involves creating multiple smaller neighbourhoods or hubs, decentralising services, and reducing reliance on cars. In larger cities or when connecting a group of minor villages, the 30-min territory focuses on improving accessibility between different neighbourhoods, surrounding suburbs, or small villages [35]. Thereby, the 15-min city is not a one-size-fits-all solution. Instead, it is a flexible planning tool that depends on the unique urban environment and the specific policy fields and instruments involved [36,37].

**Figure 2:**
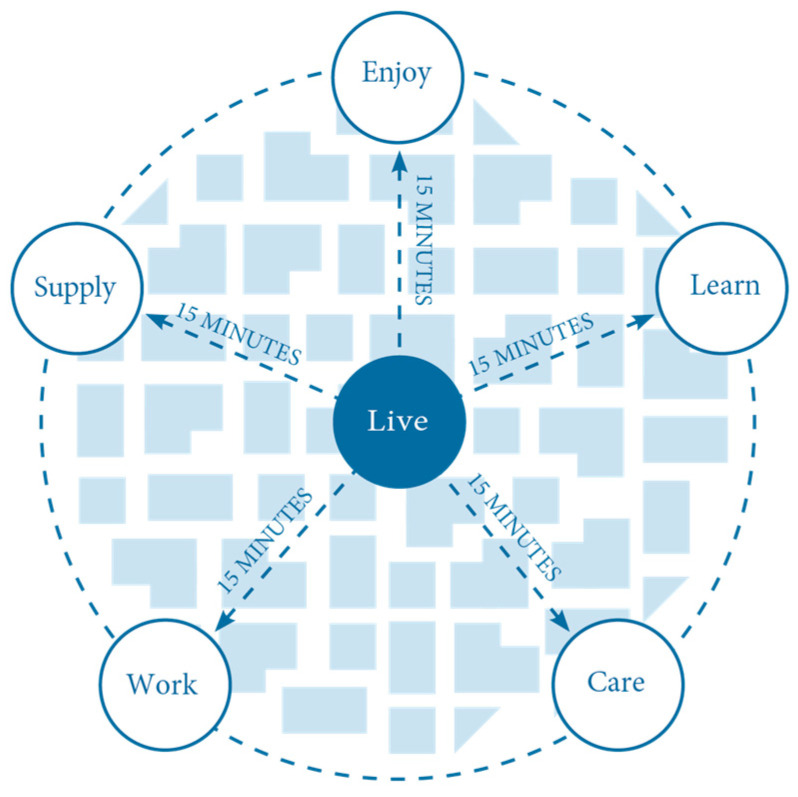
15 min city functions [40].

Even when the 15-min city’s emphasis is on localised urban living, many activities will still take place outside the 15-min city. The focus is on having proximity to everyday life functions, eliminating the need to travel longer distances for essential tasks. In this way, the growing international interest in the 15-min city reflects a paradigmatic shift away from conventional monofunctional urban planning [38]. It signals an embrace of alternative spatial strategies that prioritise human-scale accessibility, social equity, communities and sustainability [31,39]. At the core of the 15-min city model are six essential urban functions, all intended to be accessible within a 15-min walk or bicycle ride from one’s residence [33]. The six functions are presented in Figure 2. Living—inclusive and affordable housing options across socio-economic groups; Working—decentralised workplaces and remote work infrastructure; Supplying—access to goods and services through local commercial amenities; Caring—availability of healthcare facilities, including clinics and wellness centres; Learning—proximity to educational institutions, from early childhood care to higher education; and Enjoying—presence of cultural venues, green public spaces, and opportunities for leisure and social engagement, see Figure 2.

By embedding these six functions within compact urban geographies, the 15-min city challenges the legacy of modernist planning ideologies that emphasised spatial separation of land uses. Instead, it promotes a relational urbanism grounded in proximity, density, diversity, and equity [33,41]. This reconceptualisation not only supports sustainability and well-being agendas but also enhances urban resilience amid ongoing global crises.

While the temporal metric of 15 min provides a general framework, the actual distance covered is contingent upon factors such as the chosen mode of transport and the individual’s physical capabilities, which is especially relevant in relation to community health centres. The focus on proximity does not negate the importance of public transportation within the broader urban mobility system. Instead, it highlights the need to prioritise active mobility as a fundamental component for promoting public health, well-being, social cohesion, and equitable access to urban amenities [39,41]. Nevertheless, a comprehensive and efficient public transportation network remains essential, particularly for ensuring connectivity between neighbourhoods, urban districts, and regional or national destinations. In this context, active mobility and public transit are complementary pillars of a sustainable and inclusive urban mobility strategy.

## 3. Methodology

Using a qualitative, place-based approach, this study examined how accessibility affects access to and experiences of healthcare at Danish community health centres. This understanding is informed by a relational perspective on space, acknowledging that accessibility is influenced not only by physical distance but also by social, infrastructural, and institutional factors [22].

The study was conducted across 11 of the 36 community health centres in Denmark, which were carefully selected to represent a range of geographical settings, including urban, rural, and island areas (see Table 1). These centres showcase a new approach to delivering healthcare, one that is decentralised and cross-sectoral, aiming to offset the centralisation of hospital services.

Data were collected between 2022 and 2024 through semi-structured interviews [42], mobile interviews and ethnographically informed field observations.

A total of 24 semi-structured interviews were conducted with two participant groups. Nineteen healthcare professionals (including general practitioners, nurses, physiotherapists, and administrative staff) and five patients were interviewed (see Table 2). All interviews explored perceptions of geographic accessibility, care continuity, spatial barriers, and the perceived value of localised health infrastructure. Patients were explicitly asked about their experiences with travel, distance, and the importance of proximity to services.

Field visits were conducted at each community health centre to assess spatial integration, transportation access, and physical proximity to other services and community infrastructure. Additional observations included travel time by public transport and walkability assessments from nearby urban and residential zones.

All interviews were audio-recorded, transcribed verbatim, and analysed using NVivo. A reflexive thematic analysis was conducted, employing a combination of inductive and deductive coding strategies [43]. We generated initial codes based on a grounded theory approach that identified patterns in the data and then refined them using theoretical concepts such as transport needs, proximity, accessibility, and relational proximity. We triangulated observational and documentary data with the interview findings to support the analysis and place individual narratives within the broader context of policy and planning frameworks.

## 4. Rethinking Accessibility in Healthcare Through the Lens of the 15-Min City

Across the studied Danish community health centres and municipalities, accessibility emerges as an organising principle for local health services. Drawing on the aspirations behind the 15-min city paradigm [33], local actors aim to provide care close to where the citizens live. However, our data reveal the tensions and paradoxes involved in adapting this so far mostly urban-centred model to rural and island geographies, where population density, mobility infrastructure, and institutional presence are notably different. This does not entail, however, that inspiration cannot be drawn from the 15-min city concept, for instance, in relation to rethinking the role of infrastructure to facilitate active mobility instead of cars. Nevertheless, in small-island municipalities, physical distance to larger health facilities poses a significant obstacle. As patients say, the need to travel for care, especially by ferry, challenges their ability to conserve energy, stay independent, and maintain their dignity. Here, inspiration from the 15-min city concept can help develop a strategy in which care is delivered to citizens, either physically or digitally, or the infrastructure supports independent access through active mobility or public transport. However, this shift introduces new challenges related to digital literacy, trust, and organisational coordination.

This analysis shows that accessibility in healthcare includes physical, relational, and digital factors. Accessibility is about more than just distance; it also covers access, continuity, and a sense of safety, all of which can vary depending on different situations marked by geographic distribution and resource disparities. The 15-min city concept opens up the opportunity to implement this nuanced view on accessibility in healthcare, where the simplified idea of accessibility for cars is the main goal, and instead casts light on the many opportunities for active and independent mobility that are possible to integrate into existing infrastructures.

The analysis below demonstrates that healthcare accessibility in peripheral contexts extends beyond mere physical distance. It encompasses everyday life, social relationships, and institutional design. Three interconnected dimensions emerge: (1) breaking down barriers between health, planning, and social and cultural sustainability, (2) reorganising planning practices to align with patients’ daily geographies, and (3) using island contexts as critical cases that challenge traditional thinking around accessibility.

### 4.1. Breaking Down Silos: Integrating Health, Planning, and Social and Cultural Sustainability

Current methods of delivering health services in rural Denmark often reinforce a siloed perspective, where healthcare, urban planning, and social policy are treated as separate entities both institutionally and conceptually. Although health policy might mention local accessibility, and planning policy could focus on liveability or community resilience, these areas rarely intersect in practical applications. Nevertheless, local stakeholders consistently stressed the importance of adopting a more holistic and cross-sectoral approach that combines spatial, social, and cultural aspects of health.

This aligns with emerging debates on social and cultural sustainability in planning [18], which argue that well-being cannot be reduced to physical infrastructure or service provision alone. Social sustainability involves fostering trust, inclusion, and meaningful participation in shaping local environments. In contrast, cultural sustainability refers to the preservation and adaptation of local identities, values, and practices in a manner that supports community resilience [20]. In the context of rural health, these dimensions are crucial: healthcare is not only a technical service but also a social practice embedded in relationships, shared histories, and local meanings.

This reflects an awareness of structural issues but an inability to implement integrative approaches. In many municipalities, health is still often seen narrowly as a sectoral duty rather than as part of a broader spatial and cultural practice of well-being. On top of this, our urban and rural environments have been profoundly shaped by the rational planning paradigm that emerged in the post-World War II era. Planning became both a political and economic instrument, producing a doctrine for the so-called functional city. In this model, social challenges were addressed through strict functional segregation and zoning that divided the city into distinct areas for housing, work, and recreation to bring structure and coherence to urban space. Traffic, focused on the car, was conceived as the tool that could facilitate connections between these separate zones. These planning rationalities still influence planning today and have created a specific view that neglects the effect between health, accessibility and mobility. Planning is complex, and professional expertise frequently operates by simplifying and standardising complex realities in order to make them manageable and subject to intervention [44]. This tendency toward perpetuating narrow perspectives and limited problem definitions within professional circles has been described as ‘trained incapacity’ [45].

This is visible when looking at the overall vision of healthcare in Denmark and how this is embedded locally. As one practitioner explained, there is a profound difference between highly centralised healthcare models and locally embedded care:

*“Historically, the general practitioner resembles more a druid or a ‘wise woman’—someone local who not only knows the geography but also often knows the person. They can see the individual in their concrete context rather than as an anonymous symptom. In that way, the potential for truly person-centred medicine is far greater in general practice than in the rest of the healthcare system.”*

(GP)

However, frontline actors, including municipal health workers and local planners, recognise that physical accessibility alone is not enough without also addressing relational and cultural accessibility. A community health centre may be located in a geographically close area. However, if it fails to connect with the community’s social fabric or undermines local identities and networks, its impact remains limited. Socially and culturally sustainable healthcare approaches involve strengthening existing community structures, supporting informal care networks, and respecting local lifestyles.

In rural and island settings, this means that health planning must be rooted in the daily cultural landscapes of the area: the grocery shop that also functions as a gathering place, the multifunctional community health centre where health, social, and cultural activities intersect, and the local knowledge of what makes people feel safe and connected and works as community hubs [11]. This focus often means rejecting urban-focused models, such as the “15-min city”. However, lessons can still be learned from these types of planning concepts as long as they are adapted to the cultural logics of peripheral life, where mobility, identity, and belonging take different forms. This is first and foremost because of the focus on putting everyday life first and asking how active mobility can connect the essential elements of everyday life. Routinised everyday practices of cycling and walking are essential for older adults’ ability to move independently, particularly as they become physically challenged. By using the 15-min city concept, the focus is not on accessibility with cars but on independent movement through active mobility. This is also visible in the empirical work, where a lack of accessibility leads to a lot of frustration between healthcare patients and professionals.

### 4.2. Everyday Life and the Reorganisation of Planning for Accessibility

For patients in rural and peri-urban settings, the geographical context of everyday life is central. Accessibility is experienced not only in terms of travel distance but also in how health services integrate into daily routines and the rhythms of life. A healthcare professional from Municipality K explains the frustration of mismatched planning: *“We had agreed to hold a smoking cessation course in our community health centre in Sheep Town… but most of the citizens actually lived in New City or nearby, and now we were asking them all to drive south… so even though we thought we were bringing it closer, it actually became further away for most.”* (Municipality K)

Similarly, patients value even accessibility and proximity to healthcare facilities: *“If it is very close, I drive there myself. When I need to go to the hospital, I have to use the flex-transport service (subsidised transport), and I am very happy that I do not need it for the weekly training. It takes too long because you have to pick up so many different people on the way. It’s nice to be able to drive yourself.”* (Patient A)

For planners and healthcare workers, this involves reorganising services based on the patients’ lived geography rather than administrative boundaries. Accessibility should match how people move, care for others, and conserve their energy. This approach requires balancing local access with integrated care: making basic services more available locally while ensuring specialised care remains accessible via digital or hybrid solutions. The rhythm and time aspect of the 15-min planning concept is also to look into the use of building at different times of the day and year. This brings a creative approach where the canteen of a workplace or local school can be used for other events when not in use.

Bringing healthcare close to the patient is not only about physical distance. For many patients, visiting community health centres is also about leaving the house and participating in the community. With long distances, the patients very often use subsidised transport that can be complicated through economic and institutional arrangements and create new forms of exclusion: *“It’s not something we discuss openly, no. However, transport is extremely costly because it’s a flex transport, and I have to pay twice to get back again. I called, and they said you could get reimbursement when it was at the community health centre. But after a month, no, you can’t, not when the clinic is municipal. The municipality was very kind and said they completely understood my issue. They looked everywhere, but I wasn’t eligible for anything. I could just ask the nurse to come to my home, but I don’t want that.”* (Patient B)

This quote shows how fragmented policies and unclear eligibility criteria can weaken the benefits of decentralised health services. Even when services are geographically closer, institutional accessibility, the ability to actually use and afford these services, remains inconsistent. Furthermore, this emphasises how healthcare access is closely linked to autonomy, dignity and social interaction. The patient chose to attend appointments outside the home, despite the cost, because it helped her maintain a routine and independence.

These everyday negotiations reveal that planning for rural healthcare cannot rely solely on static spatial models. It must account for how transport systems, reimbursement schemes, and social expectations intersect to shape the lived experience of care accessibility. This highlights how a planning concept based on rhythms and time can open up different possibilities in planning rural healthcare.

Today, many of these challenges are focusing on digitalisation as one way to extend accessibility without relying entirely on physical infrastructure. As one regional actor notes, *“We need to approach our healthcare tasks differently, and digitalisation can help us deliver even more care directly to where citizens live, freeing us from the constraints of physical boundaries.”* (Region A). However, even if digitalisation offers many new opportunities, it does not solve the problem of social interaction, which is of great significance for overall health.

### 4.3. Islands as Critical Cases for Reimagining Accessibility

Small island municipalities, with fewer than 20,000 inhabitants, highlight the severe limitations of remote geographies. Here, accessibility cannot rely on traditional urban principles; instead, it is improvised through multifunctional hubs that combine healthcare, social, and civic services. This approach reduces travel demand and fosters social value by transforming healthcare spaces into gathering points, consistent with the 15-min planning concept.

This approach does have many benefits but can also be complicated, as described here by a municipal healthcare worker from Municipality G: *“It can be both an advantage and a disadvantage to live and work in healthcare on the island—it can feel a bit overwhelming to meet Mr Hansen in the local grocery store.”* (Municipality G). The complex intimacy of living and working on an island brings attention to the ferry as both a lifeline and a barrier. For these healthcare professionals, it means living on the island to avoid long commutes, but this makes the line between work and their own time blurry. This shows how physical accessibility intersects with emotional and social well-being.

Interestingly, the island setting also encourages innovation. Since services cannot be spread out, they are often combined in community health centres that include libraries, social services, rehabilitation, and preventive care, as well as police stations. Moreover, islands tend to be early adopters of hybrid models, which blend local physical access with targeted referrals to off-island care, supported by digital follow-up. Within the 15-min planning concept, multifunctionality is crucial for bringing services closer to citizens. As one local healthcare professional explains, *“Yes, geographically… the citizen was the focus… can we bring something closer to the citizens, more healthcare in one way or another—that is simply their main concern.”* (Municipality I). In this way, islands serve as crucial examples that both challenge and support the 15-min planning concept. Islands demonstrate that accessibility relies on working with everyday rhythms through bundling, hybridisation, and relational approaches rather than mere spatial proximity.

## 5. Concluding Remarks

Access to healthcare services remains a significant challenge for many urban and rural residents, particularly those who rely on public transportation. This issue is often reflected in patient experiences, in which difficulties reaching clinics or hospitals result in delayed or missed treatment [46,47]. The 15-min city model provides a framework for tackling these disparities by promoting equitable access to essential services—including healthcare—within a short walk or bike ride from homes. In this model, the spatial distribution of health infrastructure is prioritised, with healthcare hubs ideally located near public transit nodes and urban centres to ensure broad accessibility.

By reducing the need for long-distance travel, this approach reduces reliance on private vehicles, thereby lowering traffic congestion and transport-related emissions [48]. It also improves patients’ quality of life by sparing them the time and physical effort required to navigate fragmented healthcare systems. This is especially important for older people, individuals, and people with disabilities, who often face greater mobility constraints and are more affected by spatial inequalities in service provision. Incorporating the 15-min city concept into community health centre planning not only supports principles of urban sustainability and inclusivity but also bolsters public health resilience by ensuring that medical care is a locally embedded and readily accessible part of daily life.

This approach can reduce travel distances for patients, integrate sustainable healthcare practices, and adapt transport infrastructure to support healthcare access. Through this, community health centres can play a crucial role in making the Danish healthcare system not only resilient, accessible, and environmentally sustainable but also socially and culturally sustainable, which improves overall health. Future policies should continue to embrace proximity-based healthcare models, ensuring that healthcare remains both geographically and functionally accessible to all citizens.

## Figures and Tables

**Table 1:** List of municipalities.

Population Size	Number of Municipalities	Type of Buildings	
Small (Island) < 20.000	4	Repurposed hospital	8
Middle < 50.000	5	Newly built centre	3
Large < 100.000	2		

**Table 2:** Table of interviews.

Place	Interviews	Number of People
Municipalities (A–J)	11	15
Regions and GP (A–C, GP)	4	4
Patients (A–E)	5	5
In total	20	24

## Data Availability

The dataset is available on request from the authors.
